# The Mycotoxin Deoxynivalenol Potentiates Intestinal Inflammation by *Salmonella* Typhimurium in Porcine Ileal Loops

**DOI:** 10.1371/journal.pone.0023871

**Published:** 2011-08-31

**Authors:** Virginie Vandenbroucke, Siska Croubels, An Martel, Elin Verbrugghe, Joline Goossens, Kim Van Deun, Filip Boyen, Arthur Thompson, Neil Shearer, Patrick De Backer, Freddy Haesebrouck, Frank Pasmans

**Affiliations:** 1 Department of Pharmacology, Toxicology and Biochemistry, Faculty of Veterinary Medicine, Ghent University, Merelbeke, Belgium; 2 Department of Pathology, Bacteriology and Avian Diseases, Faculty of Veterinary Medicine, Ghent University, Merelbeke, Belgium; 3 Institute of Food Research, Norwich Research Park, Norwich, United Kingdom; Tulane University, United States of America

## Abstract

**Background and Aims:**

Both deoxynivalenol (DON) and nontyphoidal salmonellosis are emerging threats with possible hazardous effects on both human and animal health. The objective of this study was to examine whether DON at low but relevant concentrations interacts with the intestinal inflammation induced by *Salmonella* Typhimurium.

**Methodology:**

By using a porcine intestinal ileal loop model, we investigated whether intake of low concentrations of DON interacts with the early intestinal inflammatory response induced by *Salmonella* Typhimurium.

**Results:**

A significant higher expression of IL-12 and TNFα and a clear potentiation of the expression of IL-1β, IL-8, MCP-1 and IL-6 was seen in loops co-exposed to 1 µg/mL of DON and *Salmonella* Typhimurium compared to loops exposed to *Salmonella* Typhimurium alone. This potentiation coincided with a significantly enhanced *Salmonella* invasion in and translocation over the intestinal epithelial IPEC-J2 cells, exposed to non-cytotoxic concentrations of DON for 24 h. Exposure of *Salmonella* Typhimurium to 0.250 µg/mL of DON affected the bacterial gene expression level of a limited number of genes, however none of these expression changes seemed to give an explanation for the increased invasion and translocation of *Salmonella* Typhimurium and the potentiated inflammatory response in combination with DON.

**Conclusion:**

These data imply that the intake of low and relevant concentrations of DON renders the intestinal epithelium more susceptible to *Salmonella* Typhimurium with a subsequent potentiation of the inflammatory response in the gut.

## Introduction

The contamination of food and feed with mycotoxins poses a worldwide problem with an acknowledged negative effect on both human and animal health and significant economic and international trade implications. Deoxynivalenol (DON) is a trichothecene mycotoxin frequently contaminating maize and small grain cereals in temperate regions of Europe, North America and Asia [1]. The intake of DON contaminated food or feed can lead to adverse health effects in both humans and animals. For humans, a large-scale European study on the occurrence of *Fusarium* toxins and the dietary intake showed that 57% of the tested cereals samples such as wheat were positive for DON and based on the intake estimates, it is clear that the presence of trichothecenes can pose a public health concern [2], [3]. A study on the prevalence and level of urinary DON in the United Kingdom population indicated that urinary levels of DON were positively correlated with cereal intake suggesting that the European Union recommended maximum tolerable daily intake of 1 µg DON/kg BW may be exceeded. Urinary DON levels can be a valuable tool as exposure biomarker for biomonitoring in etiological studies of DON and human disease risks [4]. For human food products, the European Union sets maximum limits for DON in cereals and cereal-based products, ranging from 200 to 1750 µg/kg [5]. Anorexia, altered feed intake, reduced weight gain and immunologic alterations are associated with chronic low-doses ingestion of DON whereas acute high-dose exposure is characterized by emesis, diarrhea, vomiting and rectal bleeding. Among farm animals, pigs are considered particularly sensitive to the dietary intake of DON resulting in substantial economical losses [6].

Nontyphoidal *Salmonella* represents an important human and animal pathogen worldwide. Each year approximate 93.8 million human cases of gastroenteritis occur due to *Salmonella* Typhimurium around the world [7]. In 2009, *Salmonella* was the most commonly reported bacteriological agent of human food borne diseases in the USA, causing approximate 44% of confirmed food borne bacterial infections [8]. *Salmonella* Typhimurium is, together with *Salmonella* Enteriditis, the most common serotype associated with human nontyphoidal salmonellosis in Europe and USA [9], [10]. In pigs, clinical salmonellosis is not a common problem as *Salmonella* infections are mostly subclinical. However, these carrier pigs are an important contaminating source for the environment, other pigs and carcasses in the slaughterhouse and as such they pose a serious public health problem being the most important reservoir of *Salmonella* Typhimurium for humans [11]–[13].

Several studies already described the direct effects of the intake of DON contaminated food or feed on the gastrointestinal tract [14]–[23], however only few examined the possible link between DON ingestion and intestinal inflammation using *in vitro* intestinal cells line models [24]–[29]. The general effects of mycotoxins on the local intestinal immune response were reviewed by Bouhet and Oswald [24], whereas Mbandi and Pestka [27] examined the capacity of DON to potentiate chemokine and proinflammatory cytokine production in murine macrophages induced by killed irradiated suspensions of *Salmonella* Typhimurium, concluding that the induction of IL-1β, IL-6, TNFα and MIP-2 by the pathogen was potentiated by DON. Maresca *et al.* [26] indicated that besides the direct pro-inflammatory effect and potentiation of an existing inflammation, DON could also cause intestinal inflammation indirectly through alteration of the intestinal barrier function. Van De Walle *et al.* [28], [29] suggested that high concentrations of DON could trigger intestinal inflammation and worsen inflammation related parameters.

Recently a potential link between food-associated exposure to certain mycotoxins including DON, and the induction and/or persistence of Inflammatory Bowel Disease (IBD) in genetically predisposed patients has been suggested [25]. At realistic doses, mycotoxins are able to cause immune and intestinal alterations comparable with those involved in human chronic intestinal inflammatory diseases [24]. In addition, infections with enteric pathogens such as nontyphoidal *Salmonella* have been implicated in the etiology of IBD [30], [31].

With DON and salmonellosis being emerging issues with possible deleterious consequences for both animal and human health and with the gastrointestinal tract being the primary target for both, we aimed to test whether DON at low but relevant concentrations interacts with the intestinal inflammation induced by *Salmonella* Typhimurium using a porcine model of infection, representative of the human gastrointestinal gut [32]–[34].

## Materials and Methods

### Ethics statement

The *in vivo* experimental protocols and care of the animals were approved by the Ethical Committee of the Faculty of Veterinary Medicine, Ghent University, Belgium (EC 2010/065, May 3, 2010).

### Chemicals

DON stock solution of 1 mg/mL (Sigma-Aldrich, Steinheim, Germany) was prepared in anhydrous methanol and stored at −20°C. Serial dilutions of DON were prepared in cell medium or Luria-Broth (LB, Sigma-Aldrich), depending on the experiment.

### Bacterial strains and growth conditions

*Salmonella enterica* subspecies *enterica* serovar Typhimurium (*Salmonella* Typhimurium) strain 112910a was used as wild type strain. To obtain highly invasive late logarithmic cultures for invasion assays, 2 µL of a stationary phase culture was inoculated in 5 mL LB and grown for 5 h at 37°C without aeration.

The inocula for the intestinal loop model were prepared according to the temperature shift method for *Salmonella*. Cultures in LB were shaken at 130 rpm for 24 h at 25°C. After diluting threefold and adjusting the OD_600 nm_ to 1, the bacteria were incubated for 2 h at 37°C, with shaking at 130 rpm. The actual number of bacteria/ml was assessed by plating tenfold dilutions on Brilliant Green agar (BGA, Oxoid, Hampshire, UK).

### Cell cultures

The IPEC-J2 cell line, a non-transformed intestinal cell line, which was originally derived from jejunal epithelium of an unsuckled piglet was used for all the experiments [35], [36]. IPEC-J2 cells were cultured in Dulbecco's Modified Eagle Medium (DMEM)/Ham's F-12 (1∶1) (Invitrogen™ Life Technologies, Carlsbad, CA, USA) supplemented with 5% (v/v) FCS (Hyclone, Cramlington, England, UK), 1% (v/v) insulin/transferrin/Na-selenite (Gibco, Life Technologies, Paisley, Scotland), 1% (v/v) penicillin/streptomycin (Gibco) and 1% (v/v) kanamycin (Gibco).

### Cytotoxicity assay

IPEC-J2 cells were seeded into a 96-well plate at a concentration of 1×10^5^ cells/mL and cultured for 1 day or 21 days, representing undifferentiated proliferating and highly differentiated IPEC-J2 cells respectively, after which the cells were exposed to DON at a concentration of 0.01, 0.025, 0.05, 0.1, 0.25, 0.5, 1, 5 or 10 µg/mL during 24 h. The cells were maintained in an atmosphere of 5% CO_2_ at 37°C. To assess cytotoxicity, 150 µL of freshly prepared neutral red solution (33 mg/L in DMEM without phenol red) prewarmed to 37°C was added to each well and the plate was incubated at 37°C for an additional 2 hours. The cells were then washed two times with Hanks Buffered Saline Solution (HBSS) and 150 µL of extracting solution (ethanol/Milli-Q water/acetic acid, 50/49/1 (v/v/v)) was added in each well. The plate was shaken for 10 min. The absorbance was determined at 540 nm using a microplate ELISA reader (Multiscan MS, Thermo Labsystems, Helsinki, Finland). The percentage of viable cells was calculated using the following formula:
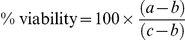
In this formula a = OD_540 nm_ derived from the wells incubated with DON, b = OD_540 nm_ derived from blank wells, c = OD_540 nm_ derived from untreated control wells.

### Invasion and translocation assays of *Salmonella* Typhimurium in porcine intestinal epithelial cells

To examine whether the ability of *Salmonella* Typhimurium to invade in and pass over a monolayer of IPEC-J2 cells was altered after pre-exposure of the IPEC-J2 cells to DON, invasion and translocation assays were performed.

For the invasion assays, IPEC-J2 cells were seeded in 24-well plates at a density of 1×10^5^ cells per well and were cultured for 1 day or 21 days depending on the experimental setup. . The 1-day-old cells were then exposed either to non-cytotoxic concentrations of DON up to 0.1 µg/mL, and to 0.25 µg/mL which decreased the percentage of viable cells to 62.3% as determined by the cytotoxicity assay described above. The 21-days-old IPEC-J2 cells were exposed to non-cytotoxic concentrations of DON up to 1 µg/mL. The invasion assay was performed as described by Boyen et al. [37]. To quantify the transepithelial passage of *Salmonella* Typhimurium over IPEC-J2 cells exposed to DON, IPEC-J2 cells were seeded on Transwell® inserts with a pore size of 3.0 µm and membrane diameter of 6.5 mm (Corning Costar Corp., Cambridge, MA) at a density of 5×10^5^ cells/mL and cultured until 21 days after reaching confluence. Cell medium was refreshed every 3 days. DON was added at concentrations of 0.1, 0.25, 0.5, 0.75 or 1 µg/mL to the apical side in 200 µL of culture medium. The basolateral side received 1 mL of blank culture medium. After 24 h of treatment with DON, the Transwell® inserts were washed three times with HBSS. Then *Salmonella* Typhimurium was added to the apical compartment at a concentration of 2×10^7^ Colony Forming Units (CFU/mL) suspended in IPEC-J2 medium with the respective concentrations of DON but without supplementation of antibiotics. The basolateral compartment was filled with antibiotic-free IPEC-J2 medium. After 60 min at 37°C and 5% CO_2_, the number of bacteria (CFU/mL) was determined in the basolateral compartment by plating 10-fold dilutions on BGA plates. Transepithelial electrical resistance (TEER) measurements were performed before and after the incubation with DON in order to evaluate the cell barrier integrity.

### Porcine intestinal ileal loop experiments

Considering the fact that the gastrointestinal tract and the immune system of pigs are very similar to that of humans [32]–[34], pig intestinal loops were used to reproduce *Salmonella* Typhimurium induced intestinal inflammation.

Two 5-week-old pigs were used in the gut loop experiments. Feed was deprived for 16 h before surgery. The pigs were anesthetized with a mixture of tiletamine+zolazepam (Zoletil 100, Virbac, Carros, France) and xylazine (Xyl-M 2%, VMD, Arendonk, Belgium), given intramuscularly at 0.2 mL/kg bodyweight after which they were intubated intratracheally. Anesthesia was maintained with 1–3% isoflurane in conjunction with 1% pure oxygen using a semiclosed circuit. Intramuscular injection of fentanyl (5 µg/kg BW/h) was used for pain management. The porcine ligated loop model was performed as described before with slight modifications [37]. After cleaning and disinfection of the abdomen, a ventral line laparotomy was performed aseptically and twelve loops were produced commencing at the distal ileum, each 6 cm in length with a short intervening segment in between. Ligation was done by an intestinal circumferential ligature through the mesentery without damaging grossly visible mesenteric arcades and thus maintaining full blood supply for both loops and inter-loop segments. A volume of 1 mL of each test condition was injected into each loop. In each pig, the treatments were randomly assigned and performed in triplicate. Following test conditions were included: negative control (LB), 1 µg/mL of DON in LB, 4×10^8^ CFU/mL of *Salmonella* Typhimurium and 1 µg/mL of DON in combination with 4×10^8^ CFU/mL of *Salmonella* Typhimurium. Piglets were maintained under anesthesia for 6 h after which they were euthanized using T61 intravenously (0.3 ml/kg bodyweight) (Intervet, Ukkel, Belgium) while still anesthetized. The pieces of the ligated intestine were then quickly excised from each loop and processed for RNA isolation.

### Analysis of intestinal cytokine response

For RNA extraction pieces of tissue samples were collected from the ileal loops, immediately frozen in liquid nitrogen and stored at −70°C until analysis. Total RNA from the intestinal samples was isolated using RNAzol®RT (MRC Inc., Cincinnati, USA) according to the manufacturer's instructions. The RNA concentration was measured by absorbance at 260 nm using Nanodrop spectrophotometer (Thermo Scientific, Wilmington, USA) and the purity of the RNA samples was checked using an Experion RNA StdSens Analysis kit (Biorad Laboratories, Hercules, CA, USA).

Reverse transcription was carried out using the iScript cDNA Synthesis Kit (Biorad Laboratories). Briefly, reverse transcription was carried out in a 20 µL final volume that included 4 µL of 5× iScript Reaction Mix, 1 µL of iScript Reverse Transcriptase, 1 µL of RNA template (1 µg), and nuclease-free water to complete the final volume. The reverse transcription mix was incubated at 25°C for 5 min, heated to 42°C for 30 min, and inactivated at 85°C for 5 min. The resultant cDNA was stored (−20°C) until further use.

Primers used for the amplification were designed using Primer3 software (available at http://frodo.wi.mit.edu/primer3) using the GeneBank sequences [38]. The primers for IL-8, TNFα and IL-12 were adopted from Volf et al. [39], [40]. The specificity of the primers was tested by performing a BLAST search against the genomic NCBI database. To optimize the amplification procedure, all primer pairs were designed to be used at the same annealing temperature (60°C). The list of genes and sequences of the primers used for quantitative PCR analysis are listed in **Table 1**. HPRT and HIS were used as housekeeping genes. Both genes had a stable expression, in all the samples tested, as calculated using the geNorm software (data not shown).

**Table 1:** List of genes and sequences of the primers used for quantitative PCR analysis.

Gene name	Forward primer (5′→3′)	Reverse primer (3′→5′)	Accession number
HPRT	GAGCTACTGTAATGACCAGTCAACG	CCAGTGTCAATTATATCTTCAACAATCAA	NM_001032376
HIS	AAACAGATCTGCGCTTCC	GTCTTCAAAAAGGCCAAC	NM_213930
IL-1β	GGGACTTGAAGAGAGAAGTGG	CTTTCCCTTGATCCCTAAGGT	NM_001005149
IL-6	CACCGGTCTTGTGGAGTTTC	GTGGTGGCTTTGTCTGGATT	M86722
IL-8	TTCTGCAGCTCTCTGTGAGGC	GGTGGAAAGGTGTGGAATGC	M86923
IL-12	CACTCCTGCTGCTTCACAAA	CGTCCGGAGTAATTCTTTGC	U08317
IL-18	ATGCCTGATTCTGACTGTTC	CTGCACAGAGATGGTTACTGC	AB010003
TNFα	CCCCCAGAAGGAAGAGTTTC	CGGGCTTATCTGAGGTTTGA	NM_214022
IFNγ	CCATTCAAAGGAGCATGGAT	GAGTTCACTGATGGCTTTGC	AY188090
MCP-1	CAGAAGAGTCACCAGCAGCA	TCCAGGTGGCTTATGGAGTC	NM_214214

To quantify the products of interest (IL-1β, IL-6, IL-8, IL-12, IL-18, TNFα, IFNγ and MCP-1) real-time quantitative PCR was utilized. The PCR reaction was carried out in 96-well plates with the appropriate forward and reverse primers (500 nM), 5 µL of iQ™ SYBR®Green Supermix (Biorad) and 1 µL of the fivefold diluted cDNA template. Thermocycling parameters were used according to the manufacturer's instructions and included 40 cycles of 20 s at 95°C, 30 s at 60°C, 30 s at 73°C. The threshold cycle values (Ct) were first normalized to the geometric means of appropriate reference mRNAs and the normalized mRNA levels were calculated according to 2^−ΔΔCt^ method [41].

### Micro-array analysis of *Salmonella* Typhimurium gene expression

To test whether DON affected the gene expression of *Salmonella* Typhimurium, a microarray analysis was performed on RNA isolated from cultures of *Salmonella* Typhimurium grown to logarithmic and stationary phase in the presence or absence of 0.250 µg/mL of DON. This concentration was chosen based on the results of the *in vitro* assays on IPEC-J2 cells.

Stationary phase cultures were obtained by aerated, overnight culture at 37°C in 5 mL LB in 50 mL flasks. To obtain highly invasive late logarithmic culture, 2 µL of a stationary phase culture was inoculated in 5 mL LB and grown for 5 hours at 37°C without aeration [42].

Of the stationary and logarithmic culture respectively, 2.00 OD_600_ units were harvested and RNA was extracted and purified using SV Total RNA Isolation Kit (Promega Benelux bv, Leiden, The Netherlands) according to manufacturers' instructions. The quality and purity of the isolated RNA was determined using a Nanodrop spectrophotometer and Experion RNA StdSens Analysis kit (Biorad). The SALSA microarrays and protocols for RNA labeling, microarray hybridization and subsequent data acquisition have been described previously [43]. RNA (10 µg) from 3 independent biological replicates of DON treated and untreated (control) logarithmic and stationary phase cultures was labeled with Cy5 dCTP and hybridized to SALSA microarrays with 400 ng of Cy3 dCTP labeled gDNA, as a common reference. Genes were assessed to be statistically significantly differently expressed between the DON treated and untreated controls at each growth phase by an analysis of variance test with a Benjamini and Hochberg false discovery rate of 0.05 and with a ≥1.5-fold change in the expression level.

### Micro array accession number

The microarray data discussed in this publication are MIAME compliant and have been deposited in NCBI's Gene Expression Omnibus [44] and are accessible through GEO Series accession number GSE29399 (http://www.ncbi.nlm.nih.gov/geo/query/acc.cgi?acc=GSE29399).

### Statistical analysis

All *in vitro* experiments were conducted in triplicate with three repeats per experiment, unless otherwise noted. The data were analyzed using Student's t-test to address the significance of difference between mean values with significance set at 0.05.

The differences in mRNA expression among groups were assessed by performing two-factor ANOVA after determination of normality and variance homogeneity. Multiple comparisons were performed using LSD. Not normally distributed data were analyzed using the non parametric Kruskal-Wallis analysis, followed by Mann-Whitney test using SPSS 17.0 Software (SPSS Inc., Chicago, IL, USA). Significance level was set at 0.05.

## Results

### DON is more toxic to undifferentiated than to differentiated IPEC-J2 cells

**Figure 1:**
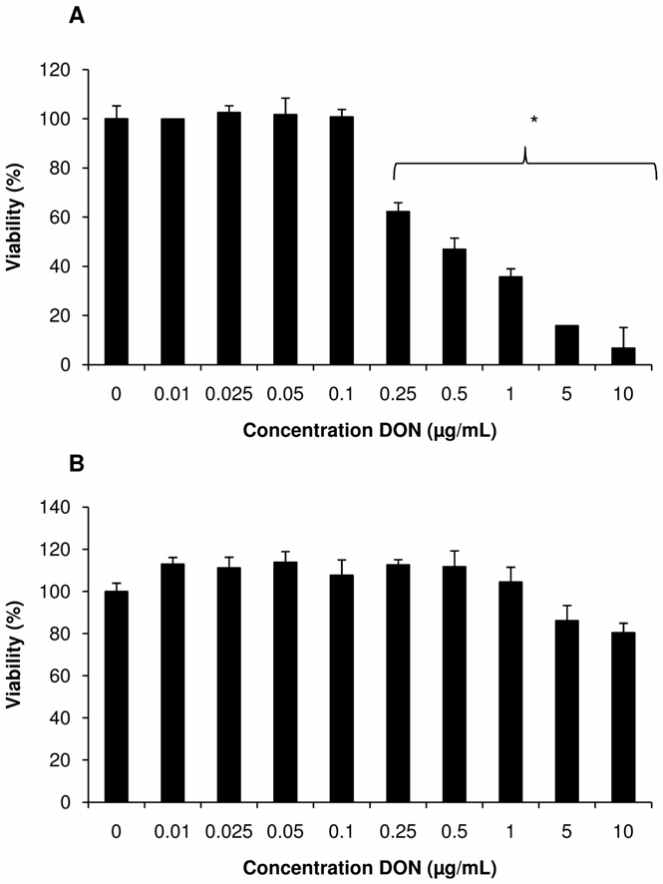
Percentage viability (%) of IPEC-J2 cells exposed to different concentrations of DON (0.005–1 µg/mL). IPEC-J2 cells were grown to a confluent monolayer during 1 day (A) or grown until differentiation during 21 days (B). Twenty-four hours after incubation with DON, the cytotoxic effect was determined using neutral red assay. Results represent the means of 3 independent experiments conducted in triplicate and their standard error of the mean. * refers to a significantly higher cytotoxic effect compared to the unexposed control cells (p<0.05).

The cytotoxic effect of DON on IPEC-J2 cells as determined using the neutral red assay is shown in **Figure 1**. The viability of 1-day-old IPEC-J2 cells was significantly decreased by exposure to concentrations of DON higher than 0.1 µg/mL (**Figure 1A**). DON concentrations up to 10 µg/mL did not significantly affect the viability of differentiated IPEC-J2 cells (**Figure 1B**). However, although not significant, exposure of differentiated IPEC-J2 cells to 5 and 10 µg/mL of DON during 24 h reduced the viability to 86+/−7% and 80+/−4% respectively.

### DON promotes the invasion of *Salmonella* Typhimurium in IPEC-J2 cells

**Figure 2:**
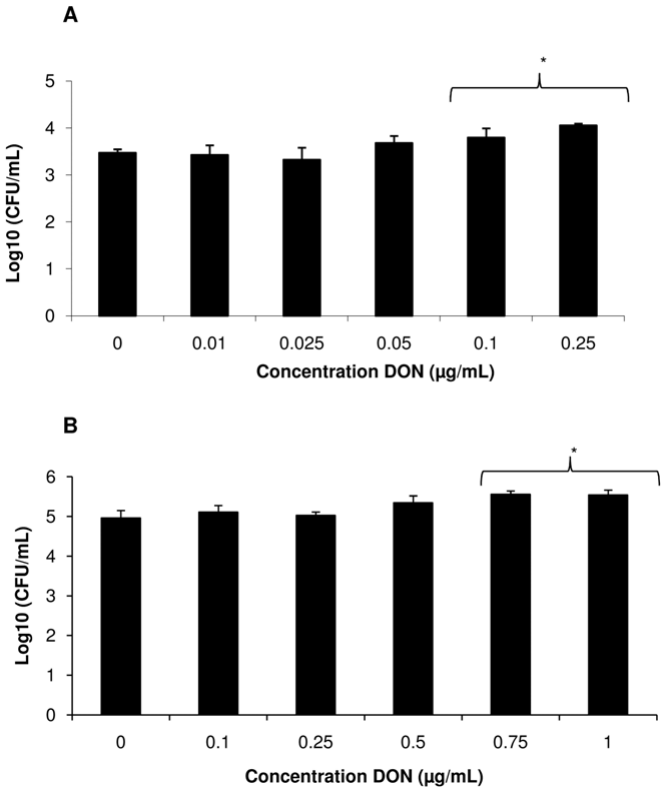
The invasiveness of *Salmonella* Typhimurium in IPEC-J2 cells after exposure to different concentrations of DON. The number of invaded bacteria in actively dividing 1-day-old (A) or differentiated 21-days-old (B) IPEC-J2 cells exposed to different concentrations of DON is shown. The 1-day-old cells were exposed either to non-cytotoxic concentrations of DON up to 0.1 µg/mL, and to 0.25 µg/mL of DON. The 21-days-old IPEC-J2 cells were exposed to non-cytotoxic concentrations of DON up to 1 µg/mL. The results show a representative of three independent experiments conducted in triplicate+standard deviation. * refers to a significantly higher invasiveness compared to unexposed control cells (p<0.05).

The results of the invasion assay with *Salmonella* Typhimurium in actively dividing IPEC-J2 cells grown are shown in **Figure 2A** whereas **Figure 2B** illustrates the invasion of *Salmonella* Typhimurium in differentiated IPEC-J2 cells.

The invasion of *Salmonella* Typhimurium was higher in undifferentiated proliferating IPEC-J2 cells exposed to concentrations of DON higher than 0.025 µg/mL in comparison to non treated IPEC-J2 cells, with a significant increased invasion when exposed to 0.1 and 0.25 µg/mL of DON during 24 h (p<0.05), although the latter (0.25 µg/mL) reduced the percentage of viable IPEC-J2 cells to 62.3%The same tendency was seen in highly differentiated 21-days-old IPEC-J2 cells, where exposure to non cytotoxic concentrations of DON led to a dose-dependent increase of the invasion of *Salmonella* Typhimurium in the cells. Exposure to concentrations of DON≥0.75 µg/mL resulted in a significantly higher bacterial count in IPEC-J2 cells compared to non treated control cells.

### DON increases the transepithelial passage of *Salmonella* Typhimurium through the intestinal epithelium

**Figure 3:**
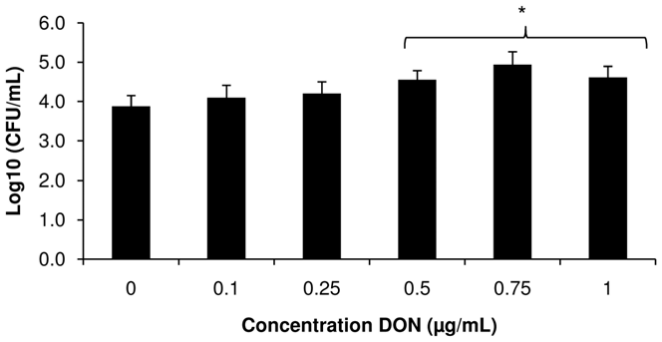
Impact of DON on transepithelial passage of *Salmonella* Typhimurium. IPEC-J2 cells seeded onto inserts until differentiation were either exposed to blank medium or treated with different concentrations of DON (0.1; 0.25, 0.5; 0.75 or 1 µg/mL) for 24 h, prior to measuring the transepithelial passage of *Salmonella* Typhimurium. The results show the means of four independent experiments conducted in triplicate with their standard error of the mean. * refers to a significantly higher translocation of the bacteria compared to the unexposed control cells (p<0.05).

We assessed the passage of *Salmonella* Typhimurium through 21-days-old IPEC-J2 cells treated for 24 h with non-cytotoxic concentrations of DON varying from 0.1 to 1 µg/mL (**Figure 3**). Exposure to DON for 24 h did not lead to a decrease in TEER (data not shown) indicating no loss of integrity of the epithelial monolayer. However, there was a significant increase in the passage of *Salmonella* Typhimurium bacteria (p<0.05) after exposure to 0.5, 0.75 and 1 µg/mL of DON in comparison to non treated IPEC-J2 cells.

### DON potentiates the intestinal inflammatory response to *Salmonella* Typhimurium in the porcine intestinal ileal loop model

**Figure 4:**
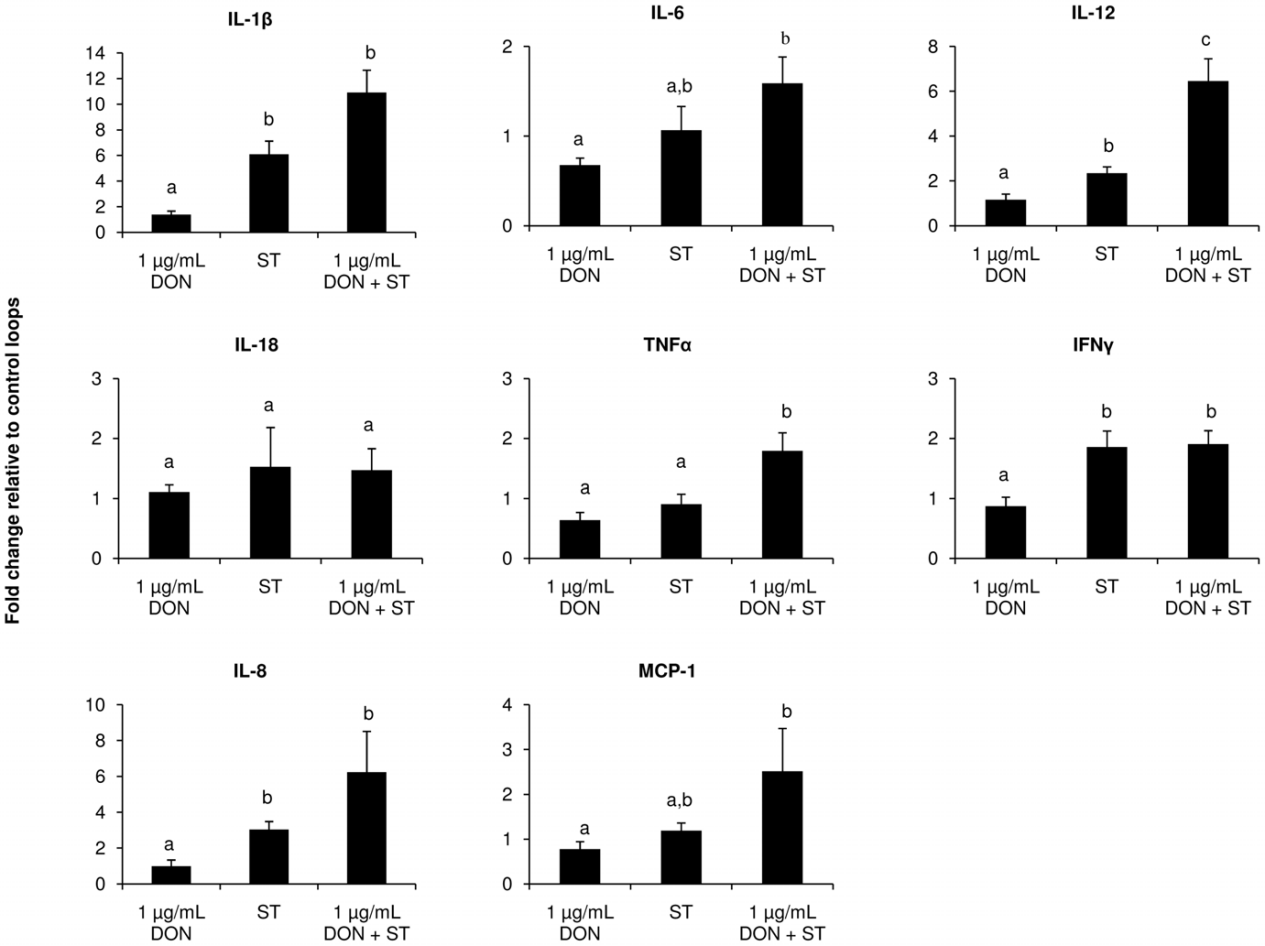
Fold change in cytokine gene expression level of the porcine ileum relative to control loops. Ileal loops were exposed to 1 µg/mL DON, *Salmonella* Typhimurium or 1 µg/mL DON+*Salmonella* Typhimurium respectively. The intestinal mRNA expression levels of the cytokines (IL-1β, IL-6, IL-12, IL-18, IFNγ and TNFα) and chemokines (IL-8 and MCP-1) 6 hours post administration was examined. Data represent the normalized target gene amount relative to control which is considered 1. Data were presented as means+standard error of the mean for a total of 6 loops per test condition. Bars marked with different letters indicate significantly different responses (p<0.05).

Using the porcine intestinal loop model, the effect of 1 µg/mL of DON, *Salmonella* Typhimurium and co-exposure of *Salmonella* Typhimurium with 1 µg/mL of DON on the intestinal mRNA expression levels of the cytokines (IL-1β, IL-6, IL-12, IL-18, IFNγ and TNFα) and chemokines (IL-8 and MCP-1) 6 hours post administration was examined. The results are illustrated in **Figure 4**. Exposure to 1 µg/mL of DON did not significantly affect the mRNA expression level of any cytokine and chemokine tested. Co-exposure of the ileal loops to 1 µg/mL of DON and *Salmonella* Typhimurium for 6 hours, resulted in a potentiated intestinal immune response in comparison to loops incubated with *Salmonella* Typhimurium but without the presence of DON. Although not significant except for TNFα and IL-12 (p<0.05), there was a higher increase in fold change in mRNA expression compared to the *Salmonella* Typhimurium inoculated loops for IL-1β (10.9 to 6.1 respectively), IL-12 (6.5 to 2.3 respectively), IL-8 (6.2 to 3.0 respectively), MCP-1 (2.5 to 1.2 respectively), TNFα (1.8 to 0.9 respectively) and IL-6 (1.6 to 1.1 respectively). However, no increase in mRNA expression level was seen for IL-18 and IFNγ.

### Gene expression of *Salmonella* Typhimurium is affected by exposure to 0.250 µg/mL of DON

In the stationary phase cultures of *Salmonella* Typhimurium, exposure to 0.250 µg/mL of DON did not affect the gene expression level of any gene tested in the microarray analysis.

In the logarithmic culture, 159 genes were significantly (p<0.05) up-or downregulated ≥1.5-fold by exposure to 0.250 µg/mL of DON (**Table S1**). A general reduction in expression of genes involved in energy production as well as an increased expression of *emrAB* multidrug efflux systems was seen. Six genes known to play a role in the pathogenesis of a *Salmonella* infection were affected by exposure to 0.250 µg/mL of DON. Three *Salmonella* Pathogenicity Island-3 (SPI-3) genes were either upregulated (*marT* and *fidL*) or downregulated (*cigR*), as well as the gene encoding for the SipA protein and *fliD* and *flgL*, two genes encoding for hook-associate proteins, which were downregulated.

## Discussion

Both DON and salmonellosis are worldwide emerging issues posing a threat for both animal and human health. To the best of our knowledge this is the first time that the combined effect of DON and an enteropathogenic pathogen such as *Salmonella* Typhimurium *in vivo* is demonstrated.

A porcine gut loop model was used to determine whether co-exposure to a physiologically relevant concentration of DON could potentiate the early intestinal immune response induced by *Salmonella* Typhimurium infection in the intestine. The choice for administering 1 µg/mL of DON was based mainly on the Commission Recommendation 2006/576/EC setting down 0.9 µg/g of DON in complementary and complete feeding stuff for pigs as guidance value [45]. Also DON concentrations in human intestine are estimated between 0.160 µg/mL and 2 µg/mL depending on the contamination level of the food [17]. Moreover, 1 µg/mL was found non-cytotoxic to differentiated IPEC-J2 cells. Undifferentiated proliferating IPEC-J2 cells are more susceptible for the toxic effects of DON compared to highly differentiated cells, as shown by the cytotoxicity assay. These results correspond with the literature describing DON to be less cytotoxic for differentiated Caco-2 cells compared to dividing cells. No cytotoxicity was observed after 24 h exposure in differentiated cells whereas IC_10_ values in the range 0.9–1.2 µM (0.260–0.356 µg/mL) were measured for dividing cells [46].

The results presented in this study provide evidence that DON at low but relevant intestinal concentrations enhances the intestinal inflammatory response to *Salmonella* Typhimurium. This is indicated by a significantly higher expression of IL-12 and TNFα (p<0.05) and a clear potentiation of the expression of IL-1β, IL-8, MCP-1 and IL-6 in loops co-exposed to 1 µg/mL of DON and *Salmonella* Typhimurium.

The enhanced intestinal inflammation may be due to a stimulation of *Salmonella* Typhimurium invasion in and translocation over the intestinal epithelium, as indicated by the results of the assays performed on both dividing and differentiated IPEC-J2 cells. It has indeed been suggested that bacterial invasion stimulates the innate pathways of inflammation by recognition of the pathogen-associated molecular patterns of *Salmonella* Typhimurium by TLRs present on epithelial cells and monocytes resulting in the production of several cytokines such as IL-8 [47].

Microarray analysis on the *Salmonella* Typhimurium gene expression revealed that exposure to DON only changed the gene expression in logarithmic phase culture of *Salmonella* Typhimurium and six genes of possible importance in the pathogenesis of a *Salmonella* infection were affected. The *marT* gene, a non essential gene for virulence, encodes for a regulatory protein and could be involved in other aspects of pathogenesis, such as chronic infection and host specificity [48]. The *fidl* and *cigR* gene products do not exhibit sequence similarity to proteins with known functions in the sequence database [48]. The genes *fliD* and *flgL* encode for proteins involved in flagellar biosyntheses [49] and the effector protein SipA has been shown to modulate actin dynamics in order to promote *Salmonella* entry into epithelial cells [50]. The general reduction in expression of genes involved in energy production indicates a possible toxic effect of DON on *Salmonella*. The increased expression of *emrAB* multidrug efflux systems suggests that these could be involved in the removal of DON out of the bacterium. No changes in gene expression were seen in the stationary phase culture of *Salmonella* Typhimurium exposed to DON, indicating that the *Salmonella* virulence was not enhanced by DON. Since none of these bacterial gene expression alterations seem to offer a clear explanation for the increased invasion and translocation of *Salmonella* Typhimurium and the subsequent potentiated inflammatory response seen after coexposure to DON, we can thus conclude that the action of DON is more likely to occur on the epithelial cells than on *Salmonella*. This enhanced intestinal inflammation may be of importance for patients genetically predisposed for IBD since both DON and *Salmonella* are mentioned as factors of potential etiological importance in the development of this chronic intestinal disorder [25], [30], [31].

The enhanced susceptibility of intestinal epithelial cells to *Salmonella* invasion and translocation is in accordance with our previous findings that concentrations of DON as low as 0.025 µg/mL enhance the uptake of *Salmonella* Typhimurium in porcine macrophages associated with ERK1/2 induced cytoskeletal reorganization [51]. Results of our present and previous studies thus indicate that DON may influence both the intestinal and systemic phase of a *Salmonella* Typhimurium infection, leading to increased host susceptibility to this pathogen.

In pigs, intake of DON contaminated feed might result in a higher infection level in the herd and consequently a higher public health risk for salmonellosis from the consumption of contaminated pork meat.

In conclusion, our results indicate that the intake of DON contaminated food or feed at realistic contamination levels and after short-term exposure, could render the intestinal epithelium more susceptible for invasive food pathogens such as *Salmonella* Typhimurium with a subsequent amplification of the inflammatory processes in the gut. Considering the frequent occurrence of DON in cereal-based foods and feeds worldwide, the importance of these findings should not be underestimated.

## Supporting Information

Table S1 **Results of micro-array analysis of** ***Salmonella*** **Typhimurium exposed to 0.250 µg/mL of deoxynivalenol.**
(XLS)
